# HIV prevalence and uptake of HIV/AIDS services among youths (15–24 Years) in fishing and neighboring communities of Kasensero, Rakai District, South Western Uganda

**DOI:** 10.1186/s12889-017-4166-2

**Published:** 2017-03-14

**Authors:** Richardson Mafigiri, Joseph K. B. Matovu, Fredrick Edward Makumbi, Anthony Ndyanabo, Doreen Nabukalu, Moses Sakor, Godfrey Kigozi, Fred Nalugoda, Rhoda K. Wanyenze

**Affiliations:** 1 Public Health Fellowship Program (PHFP) – Field Epidemiology Track, Ministry of Health-Makerere University School of Public Health, P.O. Box 7072, Kampala, Uganda; 2 School of Public Health, Makerere University College of Health Sciences, Kampala, Uganda; 3 Rakai Health Sciences Program, Kampala, Uganda

**Keywords:** HIV prevalence, Youth, Services, Care, Uptake

## Abstract

**Background:**

Although fishing communities have a significantly higher HIV prevalence than the general population, there is paucity of data on the burden of HIV and service utilization, particularly among the youth. We assessed the HIV prevalence and utilization of HIV prevention and treatment services among youth in Kasensero fishing community and the neighboring communities.

**Method:**

Data were derived from the Rakai Community Cohort Study (RCCS) surveys conducted between 2013 and 2014. The RCCS is a population-based household survey that collects data annually from individuals aged 15–49 years, resident in 48 communities in Rakai and neighboring districts in Uganda. For this analysis, socio-demographic, behavioral and HIV-related data were obtained for 792 individuals aged 15–24 years. We used logistic regression to conduct bivariate and multivariable analysis to determine the factors that are independently associated with HIV-positive status and their corresponding 95% confidence intervals. Data were analyzed using STATA version 13.

**Results:**

Overall HIV prevalence was 19.7% (*n* = 155); higher in Kasensero (*n* = 141; 25.1%) and Gwanda (*n* = 8; 11%) than in Kyebe (*n* = 6; 3.9%), *p* < 0.001 and among females (*n* = 112; 26.0%) than males (*n* = 43; 12.0%), *p* < 0.001. Uptake of HIV testing was high in both HIV-positive (*n* = 136; 89.5%) and HIV-negative youth (*n* = 435; 92%). Consistent condom use was virtually non-existent in HIV-positive youth (*n* = 1; 0.6%) compared to HIV-negative youth (*n* = 20; 4.2%). Only 22.4% (*n* = 34) of the HIV-positive youth were receiving antiretroviral therapy (ART) in 2013–2014; higher in the HIV-positive females (*n* = 31; 28.4%) than HIV-positive males (*n* = 03; 6.7%). Slightly more than half of males (*n* = 134; 53.8%) reported that they were circumcised; the proportion of circumcised youth was higher among HIV-negative males (*n* = 122; 58%) than HIV-positive males (*n* = 12; 27.9%). Factors significantly associated with HIV-positive status included living in Kasensero landing site (adjusted Odds Ratio [aOR] = 5.0; 95%CI: 2.22–13.01) and reporting one (aOR = 5.0; 95%CI: 1.33–15.80) or 2+ sexual partners in the past 12 months (aOR = 11.0; 95% CI; 3.04–36.72).

**Conclusion:**

The prevalence of HIV is high especially among young females and in landing site communities than in the peripheral communities. Uptake of HIV prevention and treatment services is very low. There is an urgent need for youth-friendly services in these communities.

## Background

Despite the general decline in new HIV infections in sub-Saharan Africa (SSA), in 2013 the region was responsible for 72% of all new HIV infections globally [1]. Thus, the HIV epidemic remains a serious public health problem globally and especially in SSA [2]. With a prevalence of 7.3%, Uganda had an estimated 1.6 million HIV infected people in 2013 [3]. HIV prevalence among individuals aged 15–24 was 3.7% and more than twice as high among females than males [4]. Youth in Uganda contribute more than 50% of the total population [5], and are thus an important population to target in HIV prevention given this high HIV prevalence.

An estimated 4.2 million people living with HIV globally are youth 15–24 years and the burden is highest among females [6]. There is concern of increase in risky sexual behaviours such as multiple sexual partners and decline in condom use among youth in many SSA countries [7, 8]. Several reasons have been advanced for this increase in risky sexual behaviors including reduced focus on primary HIV prevention in the era of antiretroviral therapy scale up [9–11]. The youth are especially vulnerable in this respect as they have limited or no historical knowledge or experience with HIV [12, 13].

Access to HIV treatment increased dramatically, from 7% in 2003 to approximately 46% of adults and 49% of children by end of 2015 [14–16]. In SSA, approximately 36.6% of those in need of treatment are able to access ART compared to 2% in 2003 [17]. However, this improvement is not equitable as the most vulnerable and high-risk groups remain grossly underserved. People living in fishing communities are among such high-risk groups that have not received adequate attention [18–20]. Uganda included fishing communities among the key population groups that were recommended for test and treat in the revised guidelines in 2013 but it is not clear if this policy shift has yielded the desired outcomes in terms of improved access to HIV treatment among this populations, and especially the young people [21].

Fishing is an important commercial enterprise and a means of survival to many Ugandans. Uganda has several lakes including Lake Victoria (the third largest Lake in the world), Lake Kyoga, George, Edward, and Albert and several large rivers (including River Nile), all of which have fishing communities and landing sites [22]. The fisheries industry was estimated to yield approximately 12% of the agricultural Gross Domestic Product (GDP) and 2.5% of the national GDP in 2011. About 1.2 million people in Uganda directly depend on fisheries industry as their main source of income [23]. An estimated 400,000 people are engaged in secondary activities such as fish processing, trading and other services and 100,000 fishermen are directly involved in fish harvest, boat and gear ownership [24]. Fishing in Kasensero communities is artisanal in nature, involving a large number of unskilled workers. A number of school going children work in the fisheries sub-sectors to earn a living [25], and 90% of fishermen are <40 years of age [26].

In Uganda, Human Immunodeficiency Virus (HIV) was first reported at Kasensero landing site in Rakai district, in the South Western region in 1982 [27, 28]. The district has continued to experience high HIV rates at 12% compared to the national average of 7.3% [27, 29]. Rakai district has a large fishing community population with identifiable high risk behaviors and limited access to health services as described in other fishing communities [19, 30, 31]. Youth in fishing communities, especially females, have a much higher HIV prevalence than youth in the general population [32]. However, most studies conducted in fishing communities have focused on risky sexual behaviors among adults and not much on access to HIV prevention and care services, especially among youth [18, 33, 34]. The objective of our study was to document HIV prevalence, risky sexual behaviors, and access to HIV prevention and treatment services, among youth in Kasensero fishing and the neighboring communities of Gwanda and Kyebe between 2013 and 2014.

## Methods

### Study design & site

This was a cross-sectional analysis of quantitative secondary data for youth aged 15–24 years who were interviewed as part of the Rakai Community Cohort Study (RCCS) surveys between 2013 and 2014. The RCCS has been described previously [35, 36]. Briefly, the RCCS is a household, population-based survey which was instituted by the Rakai Health Sciences Program (formerly Rakai Project) in 1994 for a community randomised trial for control of sexually transmitted diseases (STD) to prevent HIV. The RCCS conducts annual socio-demographic, sero-behavioral and reproductive health interviews in a cohort of approximately ~18,000 individuals aged 15–49 years, resident in 48 study communities [35]. In the 2013–2014 survey visits, adults aged 15–49 years who consented to interviews were requested to participate in RCCS. Consenting individuals were requested to provide a blood sample for HIV testing, which was done using rapid diagnostic tests (RDTs) in line with the national HIV testing algorithm [37]. The RCCS offers antiretroviral therapy at participating government health clinics within the study communities [38] and offers medical male circumcision services to non-Muslim men [39], among other HIV services.

### Study context

This paper uses data collected from Kasensero community; one of the 48 RCCS study communities in Rakai. The first HIV/AIDS cases in Uganda were identified at two landing sites of Kasensero and Lukunyu in 1982 [28]. Since then, HIV prevalence has remained high in these communities [40] but with limited access to HIV services [41]. Kasensero fishing community consists of three sub-counties of Kasensero landing site, Gwanda and Kyebe in Rakai district southwestern Uganda, approximately 200 km from the capital city Kampala. The population of approximately 18,000 people is largely mobile and depends directly or indirectly on fishing. The community includes fishermen, boat owners, fish processors, boat builders, fishing gear makers and repairers, retail traders and other casual income earners [32]. Kasensero fishing community is served by two health facilities, which offer HIV prevention, care, and treatment serves [32]. Kasensero community borders the United Republic of Tanzania to the south and Lake Victoria in the East.

### Study population

The study was conducted among 792 individual aged 15–24 years (youth) residents in Kasensero fishing community in Rakai District, southwestern Uganda. The number of youth in this analysis represents 12% (792/6406) of all the youth in the RCCS cohort. The term ‘youth’ is used to denote a period of transition from childhood and dependence to independence and adulthood, a stage that comes with several social and behavioral challenges and vulnerability [42]. Whereas the United Nations defines youth as 12–24 years, the dataset for this analysis was obtained from individuals aged 15–24 years since the RCCS cohort enrolls individuals in the age group 15–49 years [35].

### HIV diagnosis

HIV diagnosis was done using rapid HIV testing algorithm as recommended by the Ministry of Health (MoH), and confirmed by enzyme immunoassays (EIAs) for sero-incident cases. Parallel testing with Determine and Stat-Pak was conducted and reported as negative or positive if both tests were negative or positive, respectively. In case of discordance of the two tests, a third test was performed with Uni-Gold and reported as positive if it agreed with either of the two tests. A weak Uni-Gold test (weak positive) was repeated with EIA. Negative tests on second EIA were retested with *Polymerase Chain Reaction* (PCR).

### Measures

The dependent variable for our study is the HIV status and the independent variables were socio-demographic characteristics (age, sex, marital status and occupation), sexual behaviors (number of sexual partners reported in the past 12 months prior to the interview), and utilization of preventive (condom use and medical male circumcision) and care services (HIV testing, ART). Data were collected using structured questionnaires administered in private by trained same-sex interviewers. Marriage was defined as all forms of marriage including those married in church, mosque or cohabiting. Occupation was defined as any work where a participant earns a living. Three occupation categories were created, including: “fishing” (to designate those involved in the activity of fishing); “business” (selling other items at the landing site and neighboring communities, selling in shops, bars) and “other activities” which included agriculture for home and commercial use, house worker bar workers, waiter, hair dresser, construction, government and transport services. Education level was assessed based on the highest level of education attained; and was categorized as: uneducated (“none”/“no schooling”), “primary” if one had some level of primary or completed primary level at most, and “post-primary” for respondents who attained secondary and other tertiary education. The variable “illicit drug use” was used to define the use of unlawful drugs such as *marijuana*, cocaine and others. An individual was categorized as using ‘illicit drugs’ if they reported using at least one of these drugs. Alcohol use before sex was defined as drinking alcohol (beer, local brew and spirits) before sexual intercourse. Exchange of gifts for sex was defined as accepting or receiving a gift in order to have sexual intercourse. Condom use was assessed as the use of condoms: i) all the time (always), ii) some of the time or iii) never in the twelve months prior to the survey. Individuals who reported using condoms all the time with every sexual partner were considered to have used condoms consistently while those who did not use condoms with all partners all the time were categorized as “inconsistent” users. Other variables included HIV testing, use of ART among those who were HIV-positive and medical male circumcision. Medical male circumcision was assessed among non-Muslim males.

### Statistical analysis

Respondents’ characteristics were summarized and descriptive analysis was conducted to determine proportions of the different variables. Chi-square tests were performed to determine differences in proportions. Bivariate analyses were used to compute the unadjusted associations between the HIV status and independent variables including risky sexual behaviours, socio-demographic characteristics (such as education, religion, and occupation), and other variables (HIV testing and circumcision among non-Muslim males). Variables that were significant at the bivariate analysis (*p* < 0.05) were considered for the multivariable analysis. We conducted multivariable logistic regression by using variables that were significant at the bivariate analysis (communities, gender, education levels, occupation, marital status, number of sex partners and gift for sex). Backward selection into specific models was conducted for variables such as number of sexual partners, alcohol use before sex, demographic, gift for sex, occupation, and religion [43]. Analysis was performed using Stata 13 (StataCorp, College Station, TX) software.

### Ethics statement

The Science and Ethics Committee of Uganda Virus Research Institute (UVRI), the Uganda National Council of Science and Technology, and the US-based WESTERN IRB approved the RCCS.

## Results

### Characteristics of respondents

Table 1 shows the socio-demographic characteristics of the 792 individuals aged 15–24 who were included in the analysis. The mean age of the respondents was 20.5 years for both sexes. Respondents’ mean age was similar across the three communities: 19.06 ± 2.8 years in Kyebe; 20.9 ± 3 in Kasensero and 19.8 ± 2.4 in Gwanda. Slightly more than half of the respondents (54.7%, *n* = 433) were females; between 64.5 and 71.4% of the respondents had no formal education and nearly two-thirds (65%, *n* = 515) were Catholics. Fishing as an occupation was mentioned more by those living at Kasensero landing site than in the neighboring communities. A higher percentage (31%, *n* = 175) of youth in Kasensero landing site had two or more sexual partners compared to those in the neighboring communities. A higher proportion of youth (5%, *n* = 28) in Kasensero reported use of illicit drugs (heroin, marijuana and cocaine) compared to those in Gwanda and Kyebe (1%).

**Table 1:** Demographic and socio-behavioral characteristics of 792 youth by community in Kasensero fishing communities

Variables	Kasensero	Gwanda	Kyebe	*P* – Value
	*N* = 564 (%)	*N* = 73 (%)	*N* = 155 (%)	
Gender				0.310
Female	302 (54.0)	46 (63.0)	85 (55.0)	
Male	262 (46.0)	27 (37.0)	70 (45.0)	
Education				0.002
Uneducated	403 (71.4)	49 (67.1)	100 (64.5)	
Primary	120 (21.3)	19 (26.0)	53 (34.2)	
Post-primary	41 (7.3)	5 (7.0)	2 (1.3)	
Religion				<0.001
Catholic	329 (58.3)	58 (79.4)	128 (83.0)	
Protestant	80 (14.2)	7 (10.0)	7 (4.5)	
Muslim	108 (19.2)	4 (5.0)	13 (8.4)	
Other religion^a^	47 (8.3)	4 (5.5)	7 (4.5)	
Occupation				<0.001
Business	174 (30.9)	18 (24.7)	18 (11.6)	
Fishing	98 (17.4)	3 (4.1)	1 (0.7)	
Other occupation^b^	292 (51.8)	52 (71.2)	136 (87.7)	
Marital status				<0.001
Married	271 (48.0)	32 (44.0)	48 (31.0)	
Not married	293 (52.0)	41 (56.0)	107 (69.0)	
Number of sexual partners in the past 12 months				<0.001
0	77 (14.0)	20 (27.4)	68 (44.0)	
1	312 (55.3)	46 (63.0)	73 (47.1)	
2+	175 (31.0)	7 (9.6)	14 (9.0)	
Illicit Drug use				0.023
Yes	28 (5.0)	1 (1.4)	1 (1.0)	
No	536 (95.0)	72 (99.0)	154 (99.0)	
Gift for sex				0.060
Yes	19 (3.5)	1 (2.0)	0 (0.0)	
No	532 (96.5)	65 (98.5)	143 (100)	
Alcohol before sex				0.008
Yes	15 (3.0)	1 (2.0)	0 (0.0)	
No	536 (97.3)	65 (98.5)	143 (100)	

^a^Other religion includes Adventists and Saved/Born-again; ^b^Other occupation includes agriculture for home use and commercial, transport, security officer, government worker, students and bar workers

### HIV prevalence and determinants of HIV positive status among the youth

Table 2 shows the HIV prevalence among 789 youth for whom complete HIV status data were available, stratified by sex. Overall, HIV prevalence was high across the three communities (19.6%; *n* = 155); but much higher at Kasensero landing site (23.6%, *n* = 598) than in Gwanda (11%, *n* = 73) and Kyebe (3.9%, *n* = 155); *p* < 0.001. Female respondents had a higher HIV prevalence (25.9% *n* = 112) than their male counterparts (12% *n* = 43), *P* < 0.001. The higher HIV prevalence in females than males was consistent across study communities, level of education, number of self-reported sexual partners in the past 12 months and marital status. For instance, HIV prevalence among females living at Kasensero landing site was more than twice as high as that for males resident in the same locality (females: 34.4% relative to males: 14.1%; *p* < 0.001). Also, HIV prevalence among females reporting 2+ sexual partners in the past 12 months was about three times as high as that for males reporting the same number of sexual partners (females: 53.6% than males: 18.3%; *p* < 0.001). Respondents with post-primary education had a higher HIV prevalence (30%, *n* = 14) than those with primary (14.7%, *n* = 28) or no formal education at all (20.5%, *n* = 113); *P* = 0.042. Participants with two or more sexual partners had higher HIV prevalence (30.8%, *n* = 60) than those with one sexual partner (22%, *n* = 92) or those who reported no sexual partner in the past twelve months (5.3%, *n* = 3).

**Table 2:** HIV prevalence by selected socio-behavior characteristics, stratified by sex, among 789^a^ youth from Kasensero fishing community, Rakai district

Variable	Female (*N* = 431)		Male (*N* = 358)		*P*-value
	Total	*n* (%)	Total	*n* (%)	
Community					<0.001
Kyebe	85	4 (4.7)	70	2 (2.8)	
Gwanda	46	4 (8.7)	27	4 (14.8)	
Kasensero	300	104 (34.4)	261	37 (14.1)	
Education					0.042
Uneducated	289	81 (28.0)	262	32 (12.2)	
Primary	111	19 (17.1)	80	9 (11.3)	
Post-primary	31	12 (38.7)	16	2 (12.5)	
Occupation					0.032
Business	140	47 (33.6)	69	6 (8.7)	
Fishing	1	0 (0.0)	101	21 (20.8)	
Other occupation^b^	290	65 (22.4)	188	16 (8.5)	
Marital status					<0.001
Married	261	76 (29.1)	90	22 (24.4)	
Unmarried	170	36 (21.2)	268	21 (7.8)	
Number of sexual partners in the past 12 months					<0.001
0	57	3 (5.3)	108	0 (0.0)	
1	305	72 (23.6)	124	20 (16.1)	
2+	69	37 (53.6)	126	23 (18.3)	
Religion					0.662
Catholic	272	74 (27.2)	242	31 (12.8)	
Protestant	53	12 (22.6)	40	7 (17.5)	
Muslim	70	19 (27.1)	54	4 (7.4)	
Other religion^c^	36	7 (19.4)	22	1 (4.6)	
Illicit drug use					0.675
Yes	3	2 (67.0)	27	3 (11.1)	
No	428	110 (25.7)	331	40 (12.1)	
Gift for sex					0.021
Yes	6	2 (33.3)	14	6 (42.9)	
No	398	106 (26.6)	339	35 (10.3)	
Alcohol before sex					0.070
Yes	4	1 (25.0)	12	5 (41.7)	
No	400	107 (26.8)	341	36 (10.6)	

^a^Three respondents did not provide a blood sample; so, it was not possible to determine their HIV status

^b^Other occupation includes agriculture for home use and commercial, transport, security officer, government worker, students and bar workers

^c^Other religion includes Adventists and Saved/Born-again

Table 3 shows results of the bivariate and multivariable logistic regression of HIV-positive status by selected socio-demographic and behavioral characteristics. The factors that were significantly associated with HIV-positive status at the bivariate analysis included residence at Kasensero landing site, being male, being unmarried, and reporting one (1) or 2 or more (2+) sexual partners in the past 12 months. After adjusting for potential confounders, the factors that remained significantly associated with HIV-positive status included residence at Kasensero landing site when compared to residence in the neighboring communities (adjusted Odds Ratio [aOR] = 5.0, 95% CI, 2.22–13.01); having one (aOR = 5.0 (95% CI, 1.33–15.80) or two or more sex partners (aOR = 11.0 (95% CI: 3.04–36.72); being male (aOR = 0.4 (95% CI, 0.26–0.57), and being unmarried (aOR = 0.4 (95% CI, 0.27–0.56).

**Table 3:** Bivariate and multivariable logistic regression of HIV-positive sero-status and selected characteristics among 789 participants from Kasensero and surrounding fishing communities

Variable	Crude Odds Ratio (COR) and (95% Confidence Interval (CI))	*P*-value	Adjusted Odds Ratio (aOR) and (95% CI)	*P*-value
Community				
Kyebe	1.0		1.0	
Gwanda	3.1 (1.02–9.16)	0.046	2.1 (0.65–6.80)	0.211
Kasensero	8.3 (3.61–19.30)	<0.001	5.0 (2.22–13.01)^a^	<0.001
Sex				
Female	1.0		1.0	
Male	0.4 (0.26–0.57)	<0.001	0.3 (0.16–0.50)^a^	<0.001
Education				
Uneducated	1.0		1.0	
Primary	0.7 (0.42–1.05)	0.077	0.8 (0.46–1.30)	0.287
Post-primary	1.6 (0.85–3.17)	0.139	1.2 (0.57–2.63)	0.601
Occupation				
Business	1.0		1.0	
Fishing	0.8 (0.43–1.43)	0.354	1.3 (0.61–2.71)	0.520
Other occupation	0.6 (0.41–0.88)	0.011	0.9 (0.605–2.70)	0.704
Marital status				
Married	1.0		1.0	
Unmarried	0.4 (0.27–0.56)	<0.001	0.6 (0.41–0.98)^a^	0.044
Number of sexual partners in the past 12 months				
0	1.0		1.0	
1	15 (4.6–47)	<0.001	5.0 (1.33–15.80)^a^	0.017
2+	24 (7.4–78)	<0.001	11.0 (3.04–36.72)^a^	<0.001
Religion				
Catholic	1.0		1.0	
Protestant	1.0 (0.57–1.73)	1.000	0.7 (0.37–1.22)	0.190
Muslim	0.9 (0.54–1.46)	0.639	0.7 (0.407–1.20)	0.211
Other religion	0.6 (0.29–1.35)	0.233	0.5 (0.23–1.22)	0.136
Illicit Drug use				
Yes	1.0		1.0	
No	1.2 (0.51–3.30)	0.676	1.4 (0.47–3.91)	0.569
Gift for sex				
Yes	1.0		1.0	
No	0.4 (0.14–.88)	0.026	0.6 (0.22–1.70)	0.334

^a^ = significant

Table 4 shows HIV service utilization stratified by HIV status. Utilization of HIV testing services was high in both HIV-positive (*n* = 136; 87.1%) and HIV-negative youth (*n* = 435; 92%). However, regardless of HIV status, utilization of HIV testing services among females was much higher (62.7%, *n* = 358) than males (37.3%, *n* = 213). Consistent condom use was very low among both HIV-positive (*n* = 1; 0.6%) and HIV negative youth (*n* = 20; 4.2%). A higher proportion (70%, *n* = 14) of youth who were HIV-negative reported that they did not use condoms in the past twelve months compared to 30% (*n* = 6) of HIV-positive youth. Only 22.4% (*n* = 34) of the HIV-positive youth were receiving ART between 2013 and 2014; with a much higher proportion of HIV-positive females (*n* = 31; 28.4) compared to HIV-positive males (*n* = 3; 6.7%) reporting so. Slightly more than half of non-Muslim males (*n* = 134; 53.8%) reported that they were circumcised; with a higher proportion of HIV-negative males (*n* = 122; 58%) than HIV-positive males (*n* = 12; 27.9%) reporting so.

**Table 4:** HIV services uptake, stratified by HIV status, among youth who were sexually active in the last twelve months prior to the interviews (*n* = 624)

Variables	HIV positive		HIV negative		Total n (%)
	Female n (%)	Male n (%)	Female n (%)	Male n (%)	
HIV testing in last 12 months					
Yes	101 (92.7)^a^	35 (81.4)^a^	257 (96.9)	178 (85.9)	571 (91.5)
No	8 (7.3)	8 (18.6)	8 (3.02)	29 (14.0)	53 (8.5)
Condom use in last 12 months					
Always	1 (1.0)^a^	0 (0.0)	4 (1.5)	16 (7.7)	21 (3.4)^a^
Sometime	103 (94.5)^a^	42 (97.7)^a^	251 (94.7)	187 (90.3)	583 (93.4)
Never	5 (4.6)^a^	1 (2.3)	10 (3.8)	4 (1.9)	20 (3.2)
Antiretroviral therapy					
Yes	31 (28.4)^a^	3 (6.9)^a^	-	-	34 (22.4)
No	78 (71.6)^a^	40 (93.0)^a^	-	-	118 (77.6)
Medical male Circumcision					
Yes	-	12 (27.9)	-	122 (58.0)	134 (53.8)^a^
No	-	31 (72.1)^a^	-	85 (41.1)	116 (49.2)

^a^ = significant

## Discussion

In this study in which we assessed the burden of HIV and utilization of HIV prevention and treatment services, we found that nearly 20% of youth in the fishing communities were living with HIV; with a higher proportion of females living with HIV than males. The overall prevalence in these communities was much higher than the national HIV prevalence average for young people in the general population in Uganda (3.7%) [21]. HIV prevalence was much higher at the landing site than the peripheral communities. The high HIV prevalence at the landing site may be due to the migratory nature of the population based on seasonality of fish yield [44, 45]. The higher HIV prevalence among females clearly highlights the vulnerability of younger women to HIV infection [46].

One of the reasons HIV prevalence remains high in fishing communities is because the fisher-folks tend to engage in high risk sex, as evidenced by our findings in this study. In a meta-analysis and systematic review of HIV  risky sex behaviours among fishermen, Smolak [47] reported high proportions of the fisher-folk engaging in sex with more sexual partners or in transactional sex. Indeed, in our study, we found that having two or more sexual partners was significantly associated with HIV infection. In another study conducted among fishing communities, Asiki et al. [48] found low condom use with partners of known HIV status and high episodes of unprotected sex under the influence of alcohol and drugs. Underlying drivers such as high mobility, high disposable income, alcohol and drug use have been cited among the risks in this population [26]. Thus, these findings are consistent with previous studies in fishing communities and highlight the urgent need to scale up effective HIV prevention interventions among young people in these communities [18, 19]. The findings also indicate a need to pay particular attention to the young girls in relation to HIV prevention.

Consistent with other studies, HIV testing and access to treatment were higher among females than males [49, 50]. In a prior study conducted in the fishing communities of Kasensero, Lubega et al. found higher HIV services utilization among females than males and attributed this to the intensified services provision by the Rakai Health Sciences Program (RHSP) and the counselling offered at clinics [32]. However, although our study revealed higher HIV service utilization among females than males, the coverage of HIV services especially ART, is generally still below the national and global 90-90-90 targets. Coverage of prevention interventions including condom use among the sexually active and medical male circumcision among young people is also significantly below the national targets [51, 52]. A number of factors including stigma, inconvenient working hours for the highly mobile fisher-folk, and limited health infrastructure and HIV services have been cited for the low access to HIV care and prevention services in these communities [33, 53]. This is also coupled with a number of factors such as people travelling long distances to access health facilities, lack of information concerning services provided at health facilities and lack of privacy that is much needed by the youth [54]. Our findings highlight a need to review and enhance HIV service delivery among young people in these communities.

While medical male circumcision (MMC) can reduce the risk of HIV infection by up to 60% [55, 56], in our study, only about 54% of the non-Muslim youth reported that they were circumcised. Although this percentage is higher than the national average of 26%, it is still below the national target of 80% and could be further scaled up through community awareness campaigns [21, 57]. Our findings show that the proportion of circumcised youth was higher among HIV-negative males than HIV-positive males. This could be due to the protective effect of circumcision or encouragement of the negative men to circumcise for prevention.

Consistent condom use was especially very low and should be emphasized among the sexually active young people, even with the scale-up of circumcision and other prevention interventions. Fishing communities are known for high alcohol consumption which is a major driver of HIV infection [22, 45, 58, 59]. Alcohol consumption may interfere with consistent condom utilization among sexually active youth hence increasing risk of HIV infection [60]. A systematic review conducted by the World Health Organisation (WHO) reported very low use of condoms among young people in the developing countries where HIV is endemic [61]. A study done by Luciana and colleague among people with known HIV-positive status also indicated a high rate of risky sexual behaviours with inconsistent condom use [62]. Our findings suggest a need for prevention programs to integrate and enhance consistent condom use among youth in fishing communities.

### Limitation of the study

This study had a number of limitations. The data did not include young people below the age of 15 years and did not explore the barriers to service uptake. Additionally, our data did not include sexual partner type, in particular homosexual partnerships which have been associated with HIV risk among young people. These are areas for further research. However, the strengths of this study include use of up-to-date household data among young people including their risky sexual behaviors, HIV service uptake, and their HIV status data for a more objective outcome assessment. The data highlight a huge burden of HIV with suboptimal service coverage and thus an urgent need to scale access to services in this vulnerable group.

## Conclusion

There is very high HIV prevalence among youth in Kasensero fishing and the neighboring communities, especially among young females with low uptake of HIV prevention and treatment services. We recommend scale up of intensified combination of prevention interventions including awareness, condom use, male circumcision, HIV testing and treatment for HIV infected youth in fishing communities. Expansion of youth friendly services should be prioritized for these communities.

## Abbreviations

AIDS: Acquired immunodeficiency syndrome
aOR: Adjusted odds ratio
ART: Antiretroviral Therapy
CDC: Center for diseases control and prevention
CI: Confidence interval, EIA: enzyme-immunoassay
HIV: Human immunodeficiency virus
IRB: Institutional review board
MARPS: Most at risk populations
MMC: Medical male circumcision
MoH: Ministry of health
NIH: National institutes of health
RCCS: Rakai community cohort study
RDT: Rapid diagnostic test
RHSP: Rakai health sciences program
SSA: Sub-Saharan Africa
UPHFP: Uganda public health fellowship program
UVRI: Uganda virus research institute
